# Effectiveness evaluation of organized screening for esophageal cancer: a case-control study in Linzhou city, China

**DOI:** 10.1038/srep35707

**Published:** 2016-10-19

**Authors:** Qiong Chen, Liang Yu, Changqing Hao, Jinwu Wang, Shuzheng Liu, Meng Zhang, Shaokai Zhang, Lanwei Guo, Peiliang Quan, Patrick Germain, Yawei Zhang, Xibin Sun

**Affiliations:** 1 Department of Cancer Epidemiology, Henan Cancer Hospital/Institute, Affiliated Cancer Hospital of Zhengzhou University, Zhengzhou, 450008, China; 2 School of Public Health, Shanxi Medical University, Taiyuan, 030001, China; 3 Linzhou Cancer Registry, Linzhou Cancer Hospital, Linzhou, 456500, China; 4 McGill University, 845 Sherbrooke Street West, Montreal, Quebec, Canada; 5 Department of Surgery, Yale School of Medicine, 60 College Street, New Haven, 06520, USA.

## Abstract

In China, esophageal cancer has remained a large burden, and endoscopic screening is expected to reduce esophageal cancer mortality. Therefore, a population-based case-control study was conducted to evaluate the effect of screening. Cases were defined as individuals who had died of esophageal cancer, and controls were residents from the same area (three per case) who had not died of esophageal cancer, matched by gender and birth year. The exposure status (whether cases and controls had ever attended the screening or not) was acquired by inspecting the well documented screening records. A conditional logistic regression model was used to estimate the odds ratios (*OR*) and their 95% confidence intervals (95% *CI*). There were 253 cases and 759 controls. The reduction in risk of esophageal cancer mortality in individuals who had ever attended screening was 47% (*OR*: 0.53, 95% *CI*: 0.37–0.77). Compared with never-screened subjects, the *ORs* for screened subjects within 36 and 48 months before the reference date were 0.59(0.39–0.89) and 0.59(0.40–0.87); the *ORs* for 50–59 year old subjects were 0.48(0.28–0.85). The results suggest a 47% reduction in esophageal cancer mortality risk due to endoscopic screening, which may have significant implications for esophageal cancer screening in China, especially in rural areas.

There is a large regional variation in incidence of esophageal cancer worldwide. Although the incidence and mortality rates have shown a decreasing trend during the past decades in many parts of the world^1^,^2^, the incidence rate has slightly increased and the mortality rate has remained stable in China^3^,^4^. China falls within the high esophageal cancer incidence region, which is called the *esophageal cancer belt*^5^. The estimated world age-standardized incidence and mortality rates in 2012 were 12.6 per 100,000 and 10.9 per 100,000, respectively^1^.

Esophageal squamous cell carcinoma (SCC) is the most common esophageal cancer in Linzhou^6^, while adenocarcinoma is more common in Western countries, and the risk factors and natural history of these two subtypes of cancer appear to be different^7^,^8^. The development of esophageal SCC is a gradual progression from the early to the advanced stage^9^,^10^. However, because early stage esophageal cancer patients are often asymptomatic, the majority of patients in China have been diagnosed with advanced stage esophageal cancer^11^. The prognosis of esophageal cancer was closely related with the cancer stage. The five year survival rate for esophageal SCC was 70% for early stage patients according to the 7th edition of Cancer Staging Manual updated by the American Joint Committee on Cancer(AJCC7), while only 15% for advanced stage^12^. In China, the five year, ten year, and twenty year survival rates for patients discovered by screening were 86%, 75%, and 64% respectively^13^. Although the disease prognosis has improved over the past decades due to advances in diagnosis and treatment, esophageal cancer is still among the most lethal cancers. Cost benefit analysis was conducted in previous studies and it was found that screening, early diagnosis, and treatment for esophageal cancer could provide great cost savings in high-risk areas of China^14^,^15^,^16^. Early detection and diagnoses should be taken to improve esophageal cancer patients’ prognoses^17^.

Squamous dysplasia and carcinoma *in situ* have been suggested as potential targets for early detection screening because both lesions have shown high predictive power for the development of esophageal SCC in high risk populations^18^. Studies have also indicated that endoscopy with iodine staining and indicative biopsy can increase the sensitivity and specificity of esophageal cancer screening^19^,^20^. These findings have provided a potential tool for detecting esophageal cancer precursor lesions and for diagnosing early stage cases, especially in high risk populations^21^.

A national esophageal cancer screening and early treatment program has been initiated since 2005 in rural and high esophageal cancer incidence areas in China^22^. Linzhou city (the former Linxian County) lies in the east side of the Taihang Mountains with a population of 1.05 million and has been known worldwide for high esophageal cancer incidence. A population-based Cancer Registry had been established in Linzhou for decades; therefore, Linzhou city was selected as a pilot area to conduct the population based screening program. Endoscopy with iodine staining and indicative biopsy for residents aged 40–69 years old was used to identify precursor lesions and early cancer. By 2015, more than 30 thousand individuals aged 40–69 were screened in this project. We analyzed the data to investigate the effectiveness of the screening program in Linzhou, China.

## Material and Methods

The study was approved by the Institutional Review Boards at Henan Cancer Hospital and Linzhou Cancer Hospital. All methods were carried out in accordance with the approved guidelines.

### Screening Programs

The esophageal cancer screening program in Linzhou was organized and conducted by the Linzhou Cancer Hospital. Residents living in 124 villages of five towns (Hejian, Heshun, Dongyao, Hengshui and Linqi) participated in the screening program from one of the following years: 2005, 2008, 2011, 2012, or 2013. Subjects aged between 40 and 69 years old without a history of diagnosed cancer were eligible for the screening program, and about 51.2% of the target population in these villages participated in the screening program.

The endoscopic screening was carried out according to the guidelines for esophageal and gastric cancer screening, early diagnosis, and treatment program^23^. Written informed consent was acquired from all participants who participated in endoscopic screening according to the Declaration of Helsinki prior to the study. The participants were placed in the left lateral position, and the entire esophagus and stomach were visually examined by trained doctors. 1.2–1.5% iodine solution was used to stain the full length of the esophagus, and then unstained foci were targeted for biopsy. Biopsy specimens were fixed in 10% buffered formalin, embedded in paraffin, cut into 5-μm sections, and stained with hematoxylin and eosin. The biopsy slides were read by two pathologists. Participants were recalled to the clinic when early lesions were histologically diagnosed, and intervention methods appropriate to the lesions’ severity were used. For severe dysplasia/carcinoma *in situ* or intramucosal carcinomas, endoscopic mucosal resection and/or argon plasma coagulation treatments were used as local therapies. For submucosal cancers and advanced esophageal cancers, therapies included esophagectomy, radiotherapy, and other conventional treatments.

Information on participants’ demographic characteristics, life style factors, and disease history were collected using a standardized questionnaire by trained doctors in Linzhou Cancer Hospital. The individual records of participants’ information, endoscopy and pathology reports were well documented and managed by the department of cancer epidemiology of Linzhou Cancer Hospital.

### Study population

All residents aged 40–69 years in these 124 villages who did not express dissent to their records being used for evaluation purposes were targeted as the study population.

### Case selection

All of the 124 villages have been covered by a population-based cancer registry and the active and passive follow-up procedures have been routinely performed by the registry’s staff and village doctors in Linzhou. Cases were selected from the database of the Linzhou Cancer Registry, the data of which had been used in previous studies^11^,^24^. According to Morrison’s method^25^, cases were defined as individuals who had died of esophageal cancer in the 124 villages from September 2005 to December 2015. Cases were also required to meet the following criteria: 1) they must have been recorded as esophageal cancer cases during the period of September 2005 to December 2015 in the tumor registry database of the Linzhou Cancer Registry, 2) the age at diagnosis with esophageal cancer must be between 40 and 69 years old, 3) the diagnosis date must be later than the date of the screening program conducted, 4) they must have been residents in the screening area.

A total of 1609 esophageal cancer cases were identified. Among them, 710 were excluded because their ages were either less than 40 years old or over 69 years old when the screening started in their village. An additional 646 cases were excluded because the diagnosis date was earlier than the date when the screening started in their residential village. Finally, 253 cases were included in our final analysis.

### Control selection

Control subjects were selected from the resident roster which was generated from the well-documented records of the resident health documents program and the New Rural Cooperative Medical Systems operated by the local government.

Deaths were excluded from the resident roster before the control selection procedure. Three controls for each case were randomly selected from the resident roster and were matched by birth year (±2 years), gender, and residence village. The controls must be alive at the death time of their matched cases. Even if the subjects were matched, individuals under 40 years of age were excluded because they had no opportunity to be screened. A “pseudodiagnosis date” was assigned to each matched control, which was the date when that control’s matched case was diagnosed with esophageal cancer. Finally, 759 control subjects were matched with the 253 case subjects.

### Exposure measurement

The screening exposure was defined as those screens which took place before the date of cancer diagnosis in cases or the “pseudodiagnosis date” in controls. The dates of screening starting and ending in each village were extracted from the screening records. The status of whether cases and controls had ever attended the screenings or not was acquired by inspecting the screening records. The date of screening was also investigated, and age when screened was calculated, if they ever attended.

### Statistical analysis

All statistical analysis was conducted using SAS software (SAS Institute, Cary, NC). A chi-squared test was conducted to compare the difference of each characteristic between cases and controls. The odds ratios (*OR*s) and their 95% confidence intervals (95% *CI*s) were estimated using the conditional logistic regression models. The *OR*s were calculated to evaluate the associations between esophageal cancer mortality and endoscopic screening. Reference dates were the diagnosis dates for case subjects or “pseudodiagnosis dates” for controls. The *ORs* were calculated for those who had participated in endoscopic screening within 12, 24, 36, or 48 months before the reference date when the case subjects were diagnosed with esophageal cancer, compared with individuals who had never participated in any screening. The *ORs* were also calculated when the screened subjects were stratified by age (40–49 years, 50–59 years, 60 years or older). Stratified analyses were also performed by gender.

## Results

A total of 253 deceased esophageal cancer cases (156 men, 97 women) and 759 matched controls (468 men, and 291 women) were included in this study. The mean age of subjects being diagnosed with esophageal cancer was 61.8 ± 7.0 years. There were no significant differences in diagnosis age between men (61.6 ± 6.9) and women (62.1 ± 7.2). As shown in Table 1, approximately 50% of the cases were over 60 years of age. A total of 71 (28.1%) cases and 293 (38.6%) controls had ever participated in an esophageal cancer screening. Among the individuals who had ever participated in the screening, a majority of them were aged 50 years or older. The proportion of those who had participated in a screening was higher in controls than in cases for each age group.

Compared with individuals who had never participated in a screening, individuals who had ever participated in a screening program had a 47% reduced risk of dying from esophageal cancer (*OR* = 0.53 (0.37–0.77), Table 2). The *ORs* within 36 months and 48 months from the date of diagnosis were 0.59 (0.39–0.89) and 0.59 (0.40–0.87), compared with those who had never been screened before the date of diagnosis of esophageal cancer in case subjects. When examining the association by age of participating in a screening program, the *ORs* and 95% *CI* for those aged less than 50 years, 50–59 years, and 60 years or older were 1.09 (0.32–3.68), 0.48 (0.28–0.85), 0.52 (0.32–0.83) respectively.

Stratified analysis by gender was shown in Table 3. The reduction in risk of esophageal cancer mortality associated with the screening program was observed to be similar for men (*OR*: 0.54, 95% *CI*: 0.35–0.86) and women (*OR*: 0.51, 95% *CI*: 0.29–0.92). When examining the association by time before reference date and age at participating screenings, a statistically significantly reduced risk of esophageal cancer mortality was only seen in men within 36 months and 48 months before reference date (*OR* 0.55 (95% *CI*: 0.32–0.93); *OR* 0.56 (95% *CI*: 0.34–0.94) and in men who were aged 50 years and over (*OR* (95% CI): 0.46 (0.23–0.92) for 50–59 years and 0.52 (0.29–0.95) for 60 years or older).

## Discussion

Although a randomized controlled trial (RCT) is regarded as the preferred approach for evaluating cancer screening, enormous costs and huge amounts of time are required, and contamination confuses the result. Furthermore, RCT cannot be applied when screening has been widespread. The case-control study design has been regarded as an appropriate method to evaluate the efficacy of widespread cancer screening programs^26^. Several studies have evaluated the efficacy of screening programs for breast cancer, colorectal cancer, gastric cancer, and lung cancer^27^,^28^,^29^,^30^,^31^, and case control study design has been particularly effective in cervical cancer screening evaluation^32^. Few studies have evaluated the efficacy of an esophageal cancer screening program at population level. Our study reported a 47% reduction in risk of esophageal cancer mortality in subjects who participated in endoscopic screening compared with individuals who never participated in any screening. The result was consistent with a previous prospective study which showed a 55% reduction in risk of esophageal cancer mortality in an intervention group in which the subjects, screened by a one-time endoscopy examination, were compared with a control group^33^.

Endoscopy with indicative biopsy was used to screen for esophageal cancer in our study. It was also used to screen for gastric cancer in Japan and South Korea^34^,^35^,^36^. Case control studies had been conducted to evaluate the endoscopic screening effect for gastric cancer^30^. A 47% reduction in risk of esophageal cancer mortality was observed in our study; however, a smaller 30% risk reduction in gastric cancer also by endoscopic screening was found in another study^30^. The magnitude of the risk reduction of mortality due to endoscopy screening was lower in gastric cancer than in esophageal cancer. Although the screening in both gastric and esophageal cancer was conducted by endoscopy, esophagus screenings were considered to be more sensitive and specific because iodine staining could be used to identify precursor lesions^19^,^20^. The use of iodine staining and indicative biopsy could obviously increase the proportion of early diagnosed esophageal cancers and improve the subsequent treatment effect^19^,^20^.

When examining the association between screening time before the reference date and death from esophageal cancer, significant results were found within 36 months and 48 months before the reference date. The subjects of a case control study to evaluate the effect of cancer screening should be asymptomatic^25^,^26^. However, the symptomatic subjects could not be excluded in our study. The *ORs* within 12 or 24 months were higher than *ORs* within 36 or 48 months. The increased risk associated with screening within 12 months prior to the reference date is not surprising, and is likely to be a phenomenon of screen detection, which is related to lead time bias^27^,^37^. The results suggest that symptomatic subjects might be screened more often within 12 and 24 months before diagnosis because of their symptoms.

Endoscopic screening was found to benefit 50 years or older individuals in our study population, but found no difference between men and women. We didn’t find statistically significant association with screening in the 40–49 year old group, partly because almost 90% of the case subjects were aged 50 years or older, which was consistent with the incidence of esophageal cancer in Linzhou^6^.

In our study, the screening histories for cases and controls were acquired by investigating the well-documented records of the screening program. Recall bias was believed to be eliminated. However, certain limitations should also be considered when interpreting the study results.

Firstly, in studies of screening efficacy, lead-time bias occurs when the asymptomatic period in the natural history of the disease is not taken into account^38^. The only way to avoid lead-time bias is to compare actual mortality rates in the screened and unscreened populations^38^. Although a randomized controlled trial (RCT) is regarded as the preferred approach for evaluating cancer screening, enormous costs and huge amounts of time are required, and contamination confuses the result. Furthermore, the screening program in Linzhou has already been widespread for ten years, so RCT cannot be applied. Therefore, the case control study design was used to evaluate the effect for esophageal cancer screening in our study, in which case eligibility should be based on disease manifestations that develop only after lead-time intervals^25^. Cases were defined as deaths from esophageal cancer, which obviously meet this criterion.

Secondly, information on socioeconomic status, education, and income were not obtained in our study, which were known to be related with self-selection bias. Although Duffy *et al*. suggest a method correcting for non-compliance bias in case-control studies to evaluate cancer screening programs^39^, we could not adjust this bias due to a lack of previously published randomized data as an adjusting factor for esophageal cancer. Although, the screening program was conducted in rural areas of Linzhou, in which there was little difference in socioeconomic status between residents, and was freely provided to participants. These could reduce the possibility of selection bias, but it could not completely exclude confounding from risk factors correlated with socioeconomic factors. It’s one of the limitations in our study.

Thirdly, differences in the screening effect in subgroups were not observed due to the small number of cases. The screening program was also conducted in several other cities in Henan Province; however, these cities were not included in our study because the lag time after the screening program initiated in these cities was too short to observe the effect. Lastly, subjects who died of esophageal cancer within 2 years after the screening was initiated in the village were also included in the final analysis. There is a potential bias that this may magnify the screening effect.

To the best of our knowledge, this is the first population based case-control study to evaluate the effectiveness of endoscopic screening on the mortality of esophageal cancer. Our results suggested a 47% reduction in risk of esophageal cancer mortality by endoscopic screening with iodic staining and indicative biopsy. Although effectiveness in endoscopic screening for esophageal cancer was suggested, prudent interpretation is needed due to potential limitations, and future well-designed studies with larger sample sizes are needed to confirm our study results.

## Additional Information

**How to cite this article**: Chen, Q. *et al*. Effectiveness evaluation of organized screening for esophageal cancer: a case-control study in Linzhou city, China. *Sci. Rep.* **6**, 35707; doi: 10.1038/srep35707 (2016).

## Figures and Tables

**Table 1:** Distribution of case and control subjects by gender and age.

Screening factors	Men				Women				Total			
	Cases	%	Controls	%	Cases	%	Controls	%	Cases	%	Controls	%
Age												
40–49	15	9.6	43	9.2	12	12.4	38	13.1	27	10.7	81	10.7
50–59	65	41.7	195	41.7	32	33.0	94	32.3	97	38.3	289	38.1
60 years and over	76	48.7	230	49.2	53	54.6	159	54.6	129	51.0	389	51.3
Total	156	100.0	468	100.0	97	100.0	291	100.0	253	100.0	759	100.0
**χ**^**2**^	0.03				0.04				0.01			
*** P***	0.986				0.982				0.997			
Never screened	117	75.0	303	64.7	65	67.0	163	56.0	182	71.9	466	61.4
Ever screened	39	25.0	165	35.3	32	33.0	128	44.0	71	28.1	293	38.6
**χ**^**2**^	5.59				3.63				9.15			
*** P***	0.018				0.057				<0.01			
Ever screened stratified by age of participating in the screening												
40–49	5	3.2	11	2.4	4	4.1	15	5.2	9	3.6	26	3.4
50–59	16	10.3	73	15.6	16	13.4	49	16.8	29	11.5	122	16.1
60 years and older	18	11.5	81	17.3	15	15.5	64	22.0	33	13.0	145	19.1
**χ**^**2**^	6.96				3.72				9.97			
* **P***	0.073				0.294				0.019			
Ever screened stratified by time before the reference date												
Within 12 months	20	12.8	58	12.4	22	22.7	62	21.3	42	16.6	120	15.8
Within 24 months	23	14.7	84	17.9	23	23.7	76	26.1	46	18.2	160	21.1
Within 36 months	24	15.4	107	22.9*	25	25.8	91	31.3	49	19.4	198	26.1*
Within 48 months	26	16.7	114	24.4*	27	27.8	100	34.4	53	20.9	214	28.2**

*P < 0.05, **P < 0.01. The subjects who ever participated in endoscopic screening were stratified by the age (40–49 years, 50–59 years, 60 years or older) when they participated in screening and by time before the reference date (within 12, 24, 36, and 48 months before the reference date).

**Table 2:** Odds ratio of death from esophageal cancer for screened subjects compared with never screened subjects.

Screening factors	Cases	%	Controls	%	*OR*	95% *CI*
Ever attended						
Never	182	71.9	466	61.4	1	
Ever	71	28.1	293	38.6	0.53	0.37–0.77
Age of participating in the screening						
Never screened	182	71.9	466	61.4	1	
40–49 years	9	3.6	26	3.4	1.09	0.32–3.68
50–59 years	29	11.5	122	16.1	0.48	0.28–0.85
60 years and over	33	13.0	145	19.1	0.52	0.32–0.83
*P* for trend					p < 0.001	
Time before reference date						
Never screened	182	71.9	466	61.4	1	
Within 12 months	42	16.6	120	15.8	1.12	0.66–1.89
Within 24 months	46	18.2	160	21.1	0.75	0.48–1.18
Within 36 months	49	19.4	198	26.1	0.59	0.39–0.89
Within 48 months	53	20.9	214	28.2	0.59	0.40–0.87

The subjects who ever participated in endoscopic screening were stratified by the age (40–49 years, 50–59 years, 60 years or older) when they participated in screening and by time before the reference date (within 12, 24, 36, and 48 months before the reference date).

**Table 3:** Odds ratio of death from esophageal cancer for screened subjects compared with never screened subjects stratified by gender.

Screening factors	Men						Women					
	Cases%		Controls%		*OR*	95% *CI*	Cases%		Controls%		*OR*	95% *CI*
Ever attended												
Never	117	75.0	303	64.7	1		65	67.0	163	56.0	1	
Ever	39	25.0	165	35.3	0.54	0.35–0.86	32	33.0	128	44.0	0.51	0.29–0.92
Age of participating in the screening												
Never screened	117	75.0	303	64.7	1		65	67.0	163	56.0	1	
40–49 years	5	3.2	11	2.4	2.38	0.37–15.23	4	4.1	15	5.2	0.59	0.12–2.98
50–59 years	16	10.3	73	15.6	0.46	0.23–0.92	16	13.4	49	16.8	0.54	0.20–1.42
60 years and over	18	11.5	81	17.3	0.52	0.29–0.95	15	15.5	64	22.0	0.50	0.23–1.08
*P* for trend					0.007						0.030	
Time before reference date												
Never screened	117	75.0	303	64.7	1		65	67.0	163	56.0	1	
Within 12 months	20	12.8	58	12.4	1.06	0.54–2.10	22	22.7	62	21.3	1.20	0.52–2.79
Within 24 months	23	14.7	84	17.9	0.73	0.41–1.29	23	23.7	76	26.1	0.79	0.38–1.62
Within 36 months	24	15.4	107	22.9	0.55	0.32–0.93	25	25.8	91	31.3	0.66	0.35–1.25
Within 48 months	26	16.7	114	24.4	0.56	0.34–0.94	27	27.8	100	34.4	0.62	0.33–1.17

The subjects who ever participated in endoscopic screening were stratified by the age (40–49 years, 50–59 years, 60 years or older) when they participated in screening and by time before the reference date (within 12, 24, 36, and 48 months before the reference date).
